# Effect of Composition and Surface Microstructure in Self-Polarized Ferroelectric Polymer Films on the Magnitude of the Surface Potential

**DOI:** 10.3390/nano13212851

**Published:** 2023-10-27

**Authors:** Valentin V. Kochervinskii, Evgeniya L. Buryanskaya, Mstislav O. Makeev, Pavel A. Mikhalev, Dmitry A. Kiselev, Tatiana S. Ilina, Boris V. Lokshin, Aleksandra I. Zvyagina, Gayane A. Kirakosyan

**Affiliations:** 1 Laboratory of Technologies of Polymer Ferroelectrics, Bauman Moscow State Technical University, Moscow 141005, Russia; m.makeev@bmstu.ru (M.O.M.); pamikhalev@bmstu.ru (P.A.M.); 2 Laboratory of Physics of Oxide Ferroelectrics, Department of Materials Science of Semiconductors and Dielectrics, National University of Science and Technology MISIS, Moscow 119049, Russia; dm.kiselev@gmail.com; 3 A.N. Nesmeyanov Institute of Organoelement Compounds RAS, Moscow 119049, Russia; ilina.ts@misis.ru (T.S.I.); bloksh@ineos.ac.ru (B.V.L.); 4 A.N. Frumkin Institute of Physical Chemistry and Electrochemistry RAS, Moscow 119071, Russia; zvyagina.ai@gmail.ru (A.I.Z.); gayakira@mail.ru (G.A.K.); 5 Laboratory of Coordination Chemistry of Alkali and Rare Metals, N.S. Kurnakov Institute of General and In-Organic Chemistry RAS, Moscow 119991, Russia

**Keywords:** polymers, ferroelectric materials, microstructure, piezo force microscopy, self-polarization, surface potential, biomedical, technology, scientific research, electricity

## Abstract

The values of the surface potentials of two sides of films of polyvinylidene fluoride, and its copolymers with tetrafluoroethylene and hexafluoropropylene, were measured by the Kelvin probe method. The microstructures of the chains in the surfaces on these sides were evaluated by ATR IR spectroscopy. It was found that the observed surface potentials differed in the studied films. Simultaneously, it was observed from the IR spectroscopy data that the microstructures of the chains on both sides of the films also differed. It is concluded that the formation of the surface potential in (self-polarized) ferroelectric polymers is controlled by the microstructure of the surface layer. The reasons for the formation of a different microstructure on both sides of the films are suggested on the basis of the general regularities of structure formation in flexible-chain crystallizing polymers.

## 1. Introduction

The Kelvin probe force microscopy (KPFM) method of surface potential measurement is widely used in the study of ferroelectric inorganic materials. The methodological and experimental results are described in a number of papers [1,2,3,4,5,6,7,8,9,10]. It should be added to the conclusions of these papers that the relaxation processes of the polarized domain structure should take into account the noted surface potential [11]. In the case of polycrystals, it is noted that grain boundaries play a special role in the formation of the surface potential [12,13]. It is emphasized that the formed surface potential always consists of polarization and surface charge [14,15,16]. The development of this method has allowed its use in specific problems, such as the detection of graphite microchannels in diamonds [17], and, in general, it is considered promising in electrochemistry [2]. Considerably less attention is paid to the application of this method to the analysis of a relatively new class of organic polymeric ferroelectrics, which, due to the specific properties of the condensed state of chain molecules, have found application in a number of areas of engineering and biomedicine [18,19,20,21]. The specificity of the noted materials comes down to the fact that, unlike inorganic ferroelectrics, the crystal phase (which is assumed to be the source of the domains) accounts for only ~50% of the volume, while the rest of the volume is occupied by the so-called amorphous phase. This is a special condition that may be related to the “memory effect” in polymers [21,22]. The amorphous phase, having no long-range order at room temperature, is in a liquid-like state, where the cooperative mobility of chains with relaxation times of ~1 μs is realized [20]. Such a phase, having high dielectric susceptibility and mechanical compliance, leads to a non-classical mechanism of the piezo effect, whereby the main contribution is made by electrostriction and the “size effect” [18,19,20,23].

Self-polarized polymer films based on VDF and its copolymers are promising materials with multifunctional responses. Obtaining self-polarized films can significantly optimize the manufacturing processes of electronic devices. Materials based on VDF have excellent mechanical properties, chemical stability, and biocompatibility, which make them suitable materials for producing sensors, actuators, transducers, and nanogenerators [18]. Their high chemical and thermal stabilities, and the proximity of the acoustic impedance value to the value in the biological fluid, determine the prospects for their use as biomedical materials. Devices based on such materials are used to detect human physiological signals, in relation toelectrocardiograms, breath control, measuring body temperature, and pulse waves. Copolymers in the form of films, fibers, porous membranes, and three-dimensional porous frameworks are of great interest in the applications of tissue engineering for the regeneration of bones, muscles, and nerves. Composite coatings of implants are made on the basis of the VDF-TFE copolymers, which, due to their high osteoinductive properties and ability to sorb stem cells, stimulate reparative osteogenesis [20].

As shown earlier, in the polymers considered at the synthesis stage, the intrachain defects of the head-to-head (tail-to-tail) attachment of neighboring links appear in the chains, which are registered using high-resolution NMR [24,25]. In addition, the presence of oxygen-containing groups in the chains of these polymers has been observed [26]. These noted chemical defects will prevent the crystallization of the chains (ideally with one-dimensional long-range order) and should be displaced to the amorphous phase or even to the surface [24,25]. In one of the few works wherein the KPFM method was used to study thin ferroelectric polymer films [27] of small thickness, the sample was deposited on the substrate by spin coating. Such a method of obtaining films, not only made of the considered ferroelectrics but also made of other polymeric materials, immediately assumes anisotropy in the properties of both surfaces. Indeed, the crystallization of the formed film on the side of the substrate implies its contact with the substrate. The other side of the film, meanwhile, is formed (due to solvent escape) in contact with air or another gas medium. It is clear that the microstructure of both sides of the film must be different. This circumstance should be taken into account when interpreting the subsequent measurements. In the present work, this problem has been solved by studying a number of characteristics of PVDF-based films obtained in the free state. In particular, surface potential values have been measured on both sides of such films, which turned out not to be identical. The capacitance involved in the measurement scheme also includes the capacitance formed by the surface layer of the polymer. Therefore, the microstructures of both sides of the film were analyzed using the ATR IR spectroscopy technique, in which the surface layer of the film was probed. It was found that, in the case of different surface potentials of both sides of a film, the microstructures of these surfaces were not identical either.

## 2. Materials and Methods

The objects of study were commercial films of PVDF and its copolymer with hexafluoropropylene HFP, from the Arkema company (King of Prussia, PA, USA), the microstructures of which were investigated by high-resolution ^19^F NMR. The ^19^F NMR spectra of solutions of VDF/HFP in acetone-*d*_6_ were recorded with H-decoupling at 303 K on a Bruker Avance II spectrometer (Bruker Corporation, Karlsruhe, Germany) operating at a fluorine frequency of 282.48 MHz. The ^19^F NMR chemical shifts were referenced externally to CFCl_3_ (0 ppm). In addition, the process was carried out on films of a copolymer of VDF with tetrafluoroethylene (TFE), which was characterized earlier [28]. X-ray diffraction patterns were acquired with an EMPYREAN (PANalytical B.V.) diffractometer with Ni-filtered Cu Kα-radiation in Bragg-Brentano geometry. The crystallite size *l*_hkl_ in the direction normal to the hkl plane was determined using the Debye–Scherer equation:

(1) $$l_{c}=\frac{0.9k\lambda }{\cos\theta \sqrt{\beta^{2}-\beta_{et}^{2}}},$$

where *k* is the diffraction order, *λ* is the wavelength, and *β* and *β_et_* are the measured and etalon peak full-widths at half-maximum.

ATR spectra were obtained on a VERTEX 70 (Bruker Corporation, Karlsruhe, Germany) IR Fourier spectrometer in the range 4000–400 cm^−1^, using a PikeGladyATR single reflection attachment (PIKE Technologies, Madison, WI, USA) with a diamond working element. The spectra were corrected using the program included in the OPUS 7.0 software to take into account the dependence of the penetration depth of radiation into the sample on the wavelength. A surface polymer layer (0.5–2 μm thickness) was scanned. Absorption spectra were measured on a TENZOR 37 IR Fourier spectrometer (Bruker Corporation, Karlsruhe, Germany).

Topography and KPFM mappings of the polymer sample were carried out with a scanning nanolaboratory Ntegra Prima (NT-MDT SI, Zelenograd, Russia) using an NSG10/Pt (Tipsnano, Tallinn, Estonia) platinum conductive probe with a spring constant of 12 N/m. For KPFM measurements, the probe scanned the surface topography using tapping mode first and then a 1 V AC voltage was applied on the probe near its resonance frequency (~180 kHz) to measure the sample’s surface potential distribution through a DC voltage feedback loop. The scan rate was set to 0.5 Hz, and a lift scan height of approximately 50 nm was adopted. The topography and KPFM images were processed using the Gwyddion 2.63 software.

## 3. Results

**Figure 1:**
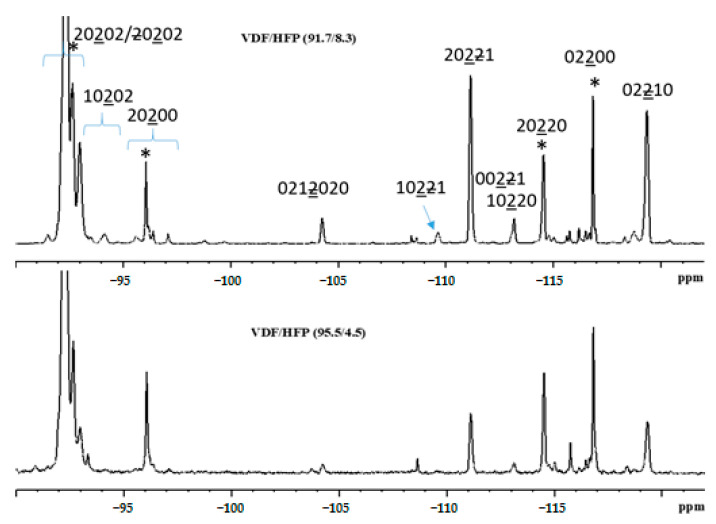
CF_2_ range of the ^19^F NMR spectra of solutions of VDF/HFP copolymers of composition 91.8/8.3 and 95.5/4.5 in acetone-*d*_6_. The signals observed in the spectrum of PVDF homopolymer are labeled with asterisks (*).

Previously [28], we studied the microstructure of a VDF/HFP copolymer containing 91.7/8.3 mol % VDF/HFP. The ^19^F NMR spectra of the copolymer studied in the present work are similar to the spectra in [29] and demonstrate that the latter contains an even lower amount of HFP units than the VDF/HFP 91.7/8.3 mol % copolymer (Figure 1). Indeed, the calculation of the composition of the copolymer under consideration via the integrated intensities of signals in the ^19^F NMR spectrum of a solution of VDF/HFP in acetone-*d*_6_ showed that the latter contained 95.5 and 4.5 mol % of VDF and HFP, respectively. Here, the authors use the same nomenclature (taken from [29]) to describe the copolymer structure, namely, 0, 1, 2, and 3. These numerals correspond to the numbers of fluorine atoms attached to a carbon atom and denote CH_2_, CF, CF_2_, and CF_3_ groups, respectively. To distinguish CF_2_ groups from VDF and HFP, 2 and 2 are used, respectively. Both comonomers (VDF and HFP) are asymmetric, and this can add to the growing chain in two ways: either as 02 (normal unit) or 20 (reverse unit) for VDF, and either 21 (normal unit) or 12 for HFP. The concentration of reverse units was calculated from ^19^F NMR spectra using the equations reported in [29]. The results of the calculation for VDF/HFP 95.5/4.5 are presented in Table 1.

The forward addition mode of VDF units leads to the usual head-to-tail linkage and produces 020202 sequences. By contrast, the reverse addition mode gives rise to the less probable head-to-head linkages 0220 and 2002. For the PVDF polymer, the content of reverse attachments has been estimated at ~5 mol %. Since the HFP units also contain the CF_2_ group, the copolymerization of VDF with HFP can be thought of as a means for introducing 22 additional defects. The contents of inverse linkages leading to 0220 and 2002 were estimated at 4.2 mol %. The most probable sequences of units in the VDF/HFP 95.5/4.5 mol % involving normally attached units like 0202022102020 contained 22 defects. Thus, 22 was the most abundant defect in the copolymer chain. In addition, the introduction of CF(CF_3_) fragments of HFP also led to the appearance of 0202210202 defects involving side CF_3_ groups.

**Figure 2:**
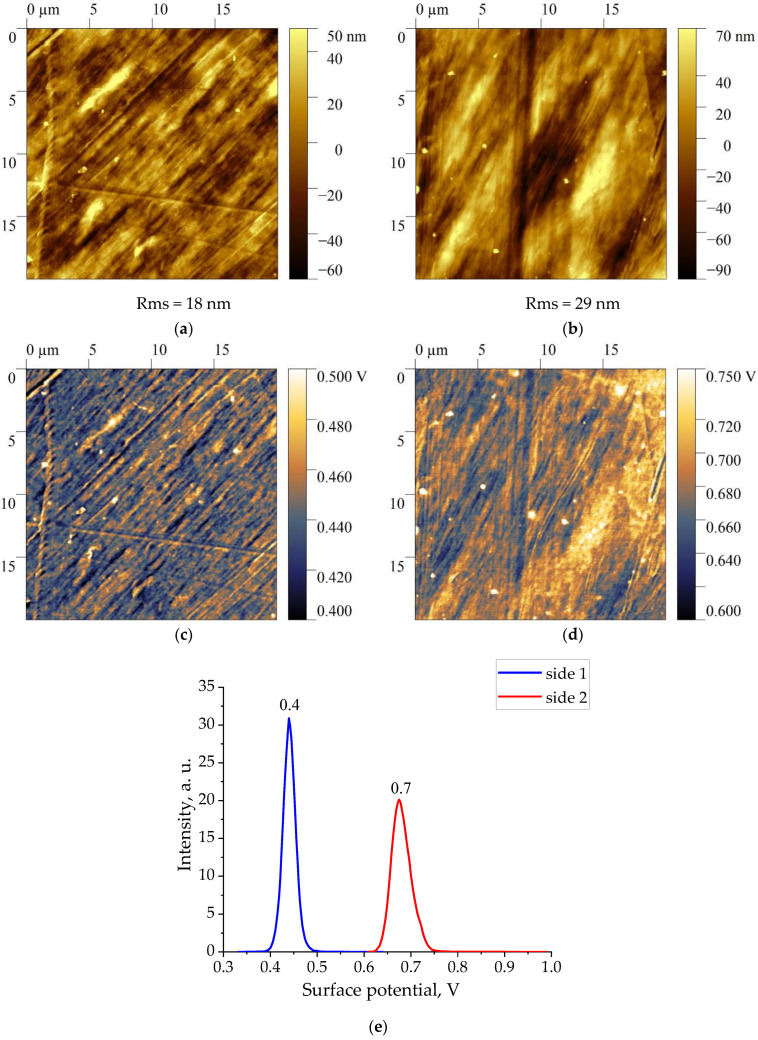
Topography (**a**,**b**), surface potential distribution (**c**,**d**) and histogram of signal KPFM (**e**) on both sides of a vinylidene fluoride-tetrafluoroethylene copolymer film of composition 94/6.

**Figure 3:**
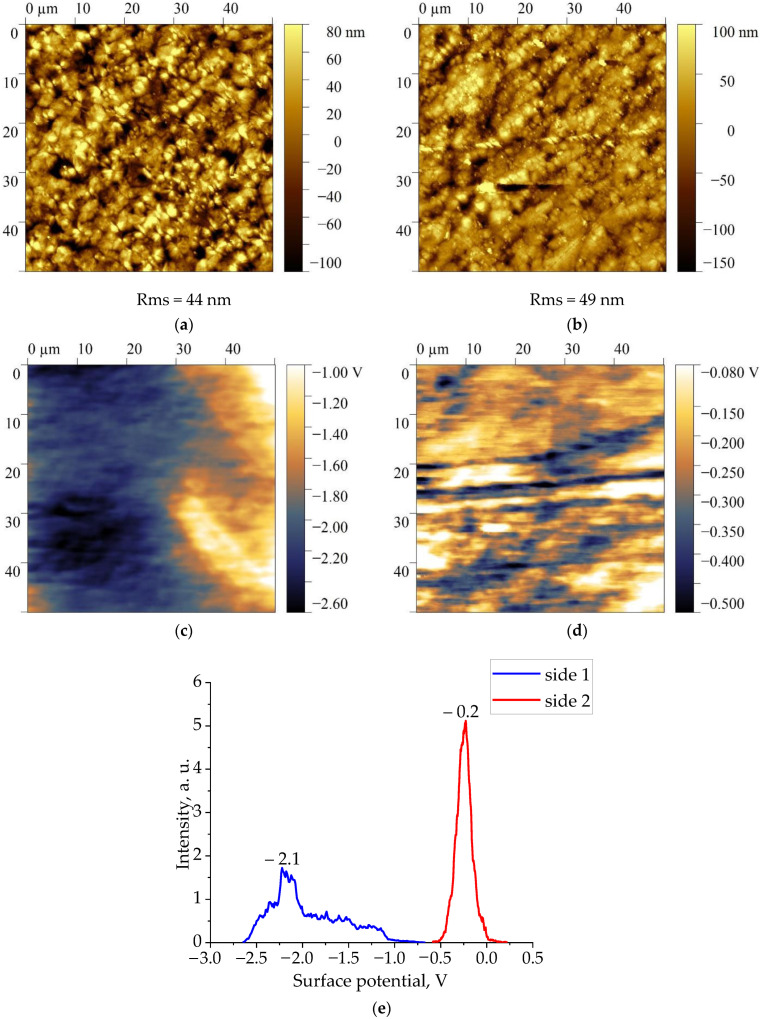
Topography (**a**,**b**) and surface potential distribution (**c**–**e**) on two sides of PVDF film.

**Figure 4:**
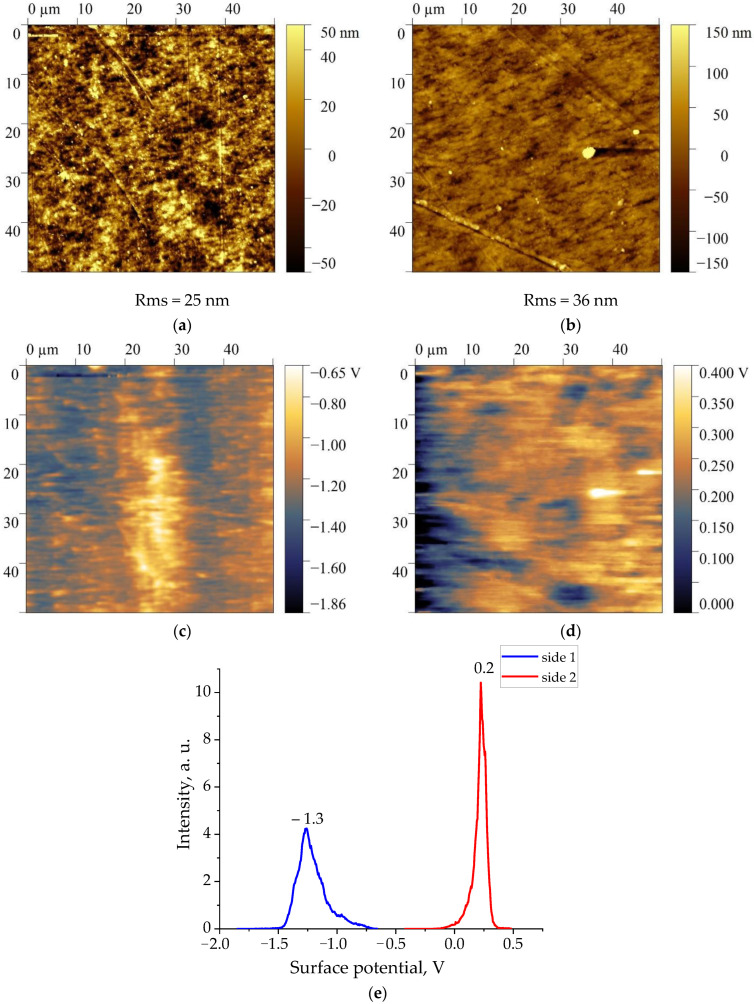
Topography (**a**,**b**) and surface potential distribution (**c**–**e**) on both sides of the vinylidene fluoride-hexafluoropropylene copolymer film of composition 96/4.

**Figure 5:**
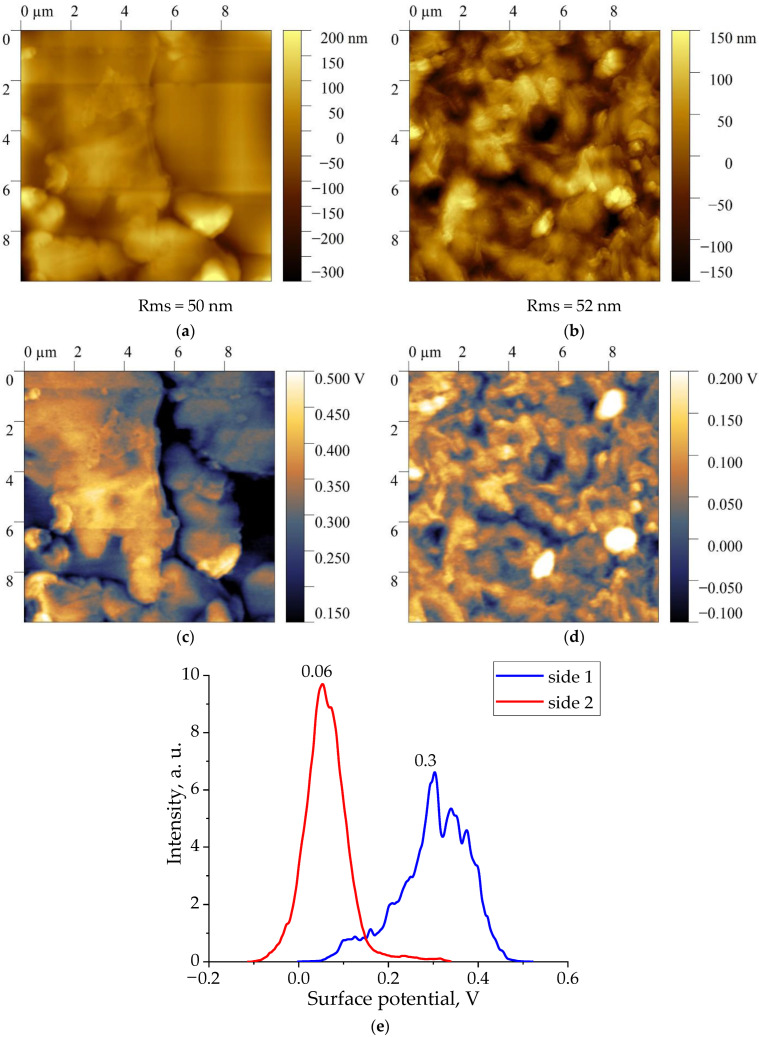
Topography (**a**,**b**) and surface potential distribution (**c**–**e**) on both sides of the vinylidene fluoride-hexafluoropropylene copolymer film of composition 92/8.

Figure 2, Figure 3, Figure 4 and Figure 5 show the topographies and surface potential values on both sides of the surfaces of all four films studied.

The analysis shows that the values of the surface potential in all films are different from zero. Since the samples were not subjected to polarization beforehand, the authors believed that self-polarization processes took place during their crystallization [30,31,32,33]. As shown in Figure 2, Figure 3, Figure 4 and Figure 5, the values of the surface potential on both sides of the films differ markedly, up to the change of its sign. For polymeric ferroelectrics, such a fact has not been previously noted by anyone, and therefore the details of the observed phenomenon were closely observed. The practical use of the considered biocompatible materials in medicine [20] also forced some authors to explore the mechanism of surface potential formation, which can affect the vital activity of the body’s cells that are in contact with the film [21]. Since the structure of the class of crystalline ferroelectric polymers (and, accordingly, domain) considered essentially depends on the thermal prehistory of the crystallization process [34], it is reasonable to give some structural characteristics of the investigated materials.

**Figure 6:**
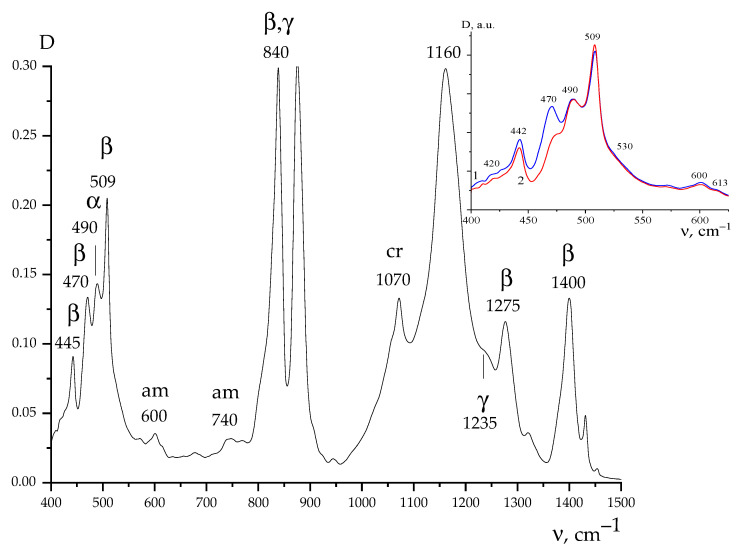
ATR spectrum obtained from one side of the VDF/TFE copolymer film of composition 94/6; inset—comparison of the ATR spectrum for the 1 and 2 sides of the film.

It is convenient to start with the VDF/TFE copolymer, the film spectrum for which is presented in Figure 6. It shows that crystallization occurs predominantly in the β-phase with planar zigzag conformation, although isomers with the conformation T_3_GT_3_G^−^ (γ-phase) and TGTG^−^ (α-phase) are also present in some amount. It can be seen from the inset to Figure 6 that the ratio of the intensities of the 470 and 490 cm^–1^ bands on both sides of the film differs quite significantly. Such data are presented in more detail in Table 2.

Therefore, in the case of the copolymer films under consideration, the ratios of intensities and other conformationally sensitive absorption bands on both sides of the film also differ. As could be seen from Figure 2 and the above table, the surface potentials on these sides also differ. Such coregulation seems to us not accidental, and therefore research has been carried out on three more types of films containing the considered ferroelectric polymers. This seems appropriate for the following reason. As noted in our case, the sign of the surface potential on both sides of the film is positive. On the other hand, the films of copolymers of VDF with trifluoroethylene, which also crystallized in the polar phase, always had a negative potential [27].

**Figure 7:**
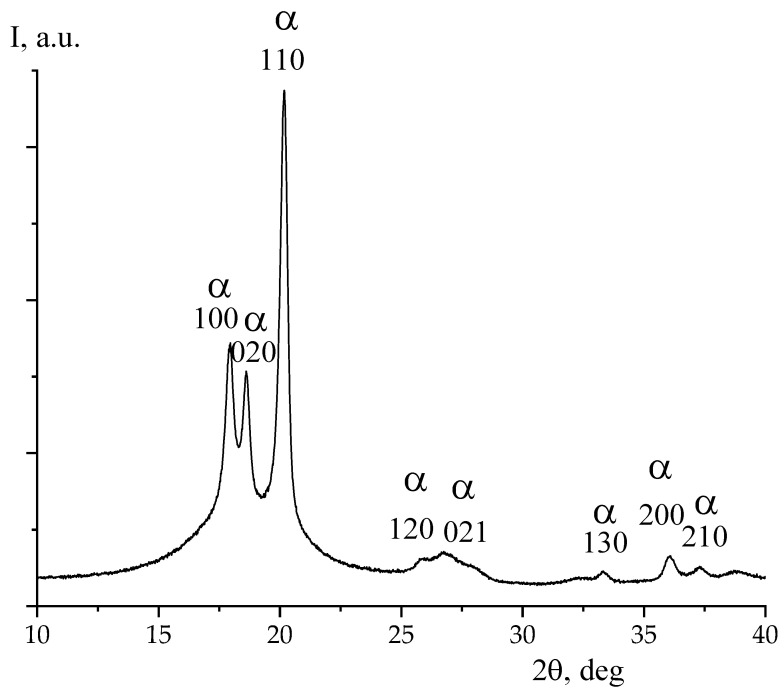
X-ray diffraction from PVDF film.

**Figure 8:**
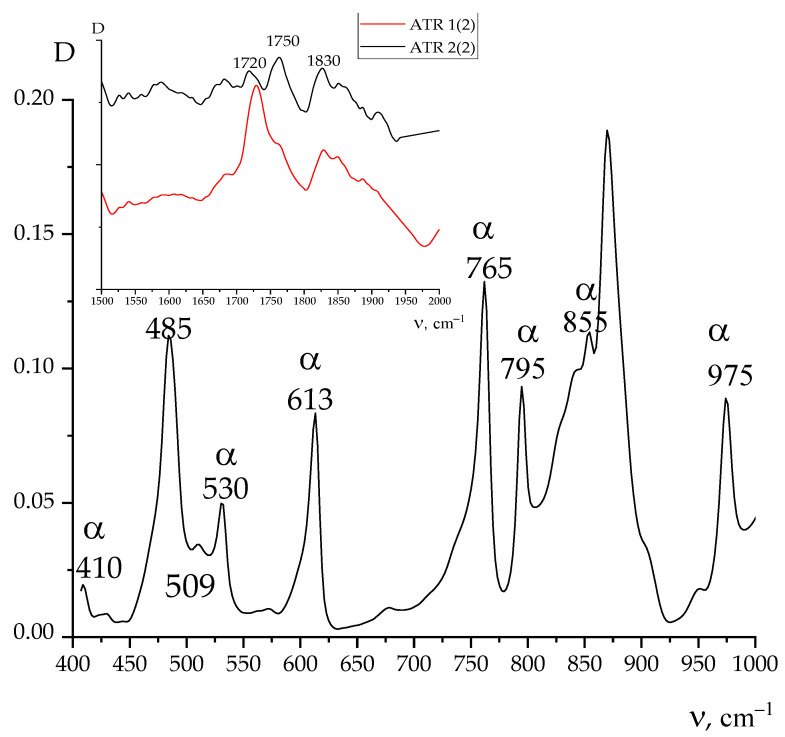
ATR IR spectrum for one side of the PVDF film; inset—comparison of ATR spectra of two sides of the film in the region of manifestation of chemical defects of PVDF chains.

Figure 7 shows X-ray diffraction and ATR IR spectroscopy data for one side of the Arkema PVDF film. From the presented data, it is evident that the above film crystallizes in the nonpolar α-phase. The same conclusion can be derived from the data of IR-spectroscopy (Figure 8). As can be seen in Figure 3 and Table 3, unlike the PVDF/TFE copolymer film, the potential on both sides of the film was negative. This means that the sign of the surface potential cannot be related only to the type of polymorphic modification of crystals in the film. Irrespective of this circumstance, Table 3 shows that the ratios of intensities of a number of characteristic bands for both sides of the film, as well as the values of their surface potential, differ.

**Figure 9:**
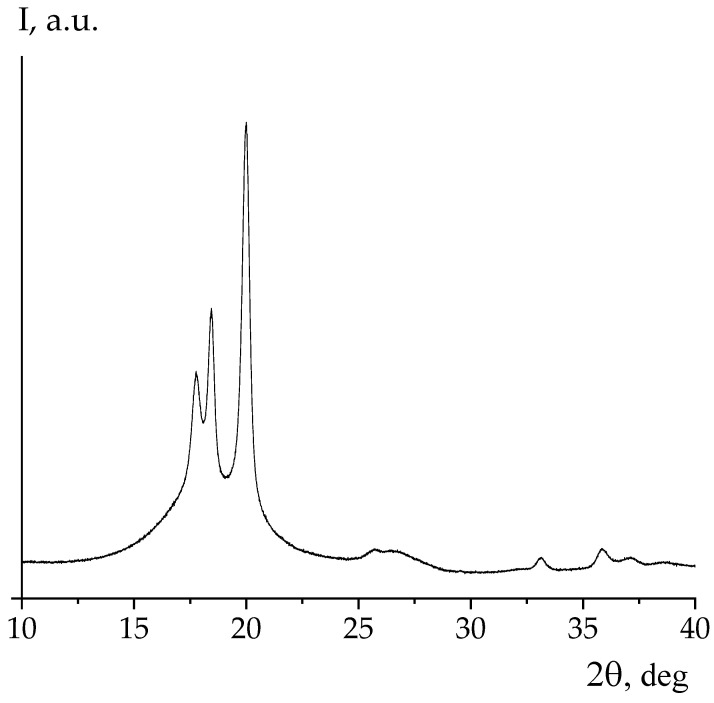
X-ray diffraction for the VDF/HFP copolymer film of composition 96/4.

Given the similar data obtained earlier on the copolymer of VDP with hexafluoropropylene HFP of composition 92/8 [28], it is interesting to trace the features of the observed regularities on the same copolymer, but with a different co-monomer ratio. For this purpose, authors have used the Arkema copolymer VDF/HFP, whose microstructure and component ratio we obtained from high-resolution 19F NMR data. From these data, it can be observed that the above film contains 4 mol % HFP, and also crystallizes in the nonpolar α-phase. For this conclusion, it is sufficient to compare Figure 7 and Figure 9, where the same reflexes are observed.

## 4. Discussion

As noted above, the type of polymorphic modification of crystals does not determine the sign of the surface potential of the film (Table 3). Indeed, for the VDP/HFP copolymer, the sign of the potential on one side of the film is negative, while on the other side it is positive. It could be thought that there are subtle details of the polymer structure that affect the sign of the surface potential. The general equation for the electrostatic force acting between the cantilever (to which we applied only an alternating voltage of frequency ω) and the charged surface of the polymer film is given below [4],

*F_es_* = 1/2*ΔV*^2^*dC(z)*/*dz*, (2)

where *z* is the normal direction with respect to the cantilever surface, and *V*, if we limit ourselves to measuring only at the first harmonic, has the following form:

*ΔV* = *V_ac_sin ωt*, (3)

The total capacitance *C* in Equation (2) could be written in the form of

*C_tot_* = *C_s_* + *C_p_* + *C_inj_*, (4)

where *C_s_*—capacitance of the surface layer of the film with the charge on it, obtained due to the self-polarization process, *C_p_*—capacitance initiated by the polarization process, and *C_inj_*—capacitance resulting from the injection of carriers from the electrodes into the polymer.

As noted in the studied case, the films were not pre-polarized, and therefore *C_p_* = 0. The amplitude of the alternating voltage *V_ac_* was 1 V, and therefore for films with a thickness of 20–50 μm, the phenomena of carrier injection can be neglected. In this case *C_tot_ = C_s_*; therefore, for the interpretation of the data on the surface potential of both sides of the film, it is possible to refer to the results of ATR IR spectroscopy for these sides. In this case, the authors can associate the microstructure of the surface layer with the value of its dielectric permittivity.

It should be recalled that the marked surface will contain a mixture of chain segments in different isomeric states. For PVDF and its copolymers, three conformations are commonly referred to—(1) planar zigzag, (2) TGTG^−^ and (3) T_3_GT_3_G^−^—which are characteristic of *β*- (polar cell), *α*- (non-polar cell) and *γ*-phase (polar cell) crystals, respectively [18,19,20]. In the IR spectra of the considered polymers, we can also identify bands that are responsible for the amorphous phase: 600, 740 and 905 cm^−1^. This is an important remark, since its chains at room temperature are in a liquid-like state, where the dielectric permittivity is quite high.

In view of the above, it is possible to refer once again to Table 2, which shows comparative data for a number of absorption bands of the VDF/TFE copolymer film. It shows that the increase in the proportion of *β*-phase isomers relative to those in the *γ*- or *α*-phase for different sides of the film is accompanied by a decrease in the surface potential. From the point of view of the previously mentioned, this can be explained by the fact that the transverse component of the dipole moment of the link in the planar zigzag conformation is higher than those for the TGTG^−^ and T_3_GT_3_G^−^ conformations. Due to this, the dielectric constants of the layers (and as a consequence, the capacitance) on different sides of the film are unequal, which leads to the difference in their surface potentials (Table 2). The reasons for this may be related to the non-identical crystallization conditions of both sides of the film [34] during its forming.

It could be seen from the second column of Table 3 that for PVDF and its copolymer with HFP films, the change in the ratios of rotational isomers in their surface will also affect the value of the surface potential. From the same table, one could infer that there are additional factors that can affect the capacitance of the polymer surface layer. The inset to Figure 8 shows the region of the spectrum where PVDF has no intrinsic absorption bands [18,19,20]. The highlighted absorption bands are associated with the presence of chemical defects in the polymer chains in the form of a small number of oxygen-containing (carbonyl or carboxyl) groups [26]. Such groups, without long-range order, reduce the packing density of chains in the surface layer, changing the dielectric constant and, accordingly, its capacitance. It was shown earlier that such defects are displaced into the surface during film crystallization, the chemical composition of which was controlled by X-ray photoelectron spectroscopy [26]. Another type of intrachain defect in the polymers under consideration is the head-to-head (tail-to-tail) attachment of neighboring links. Their tendency towards aggregation and displacement into the surface was found earlier on a number of VDF copolymers [24,25]. This was proven by the fact that a second doublet appeared in the ATR spectra in the region of valence vibrations of methylene groups, for which the frequency position (2850 and 2920 cm^−1^) coincided with that of polyethylene [24]. It was proposed to consider the relative fraction of such defects in the surface via the ratios of the intensities of the 2920/3024 cm^−1^ antisymmetric vibration bands. Such ratios for the two objects of study are summarized in Table 2. Data on the VDF/HFP copolymer of composition 92/8 have been published previously [28], and therefore, it seems reasonable to compare some structural characteristics of this copolymer with those of the polymers considered in this work, which also crystallize in the nonpolar *α*-phase.

**Figure 10:**
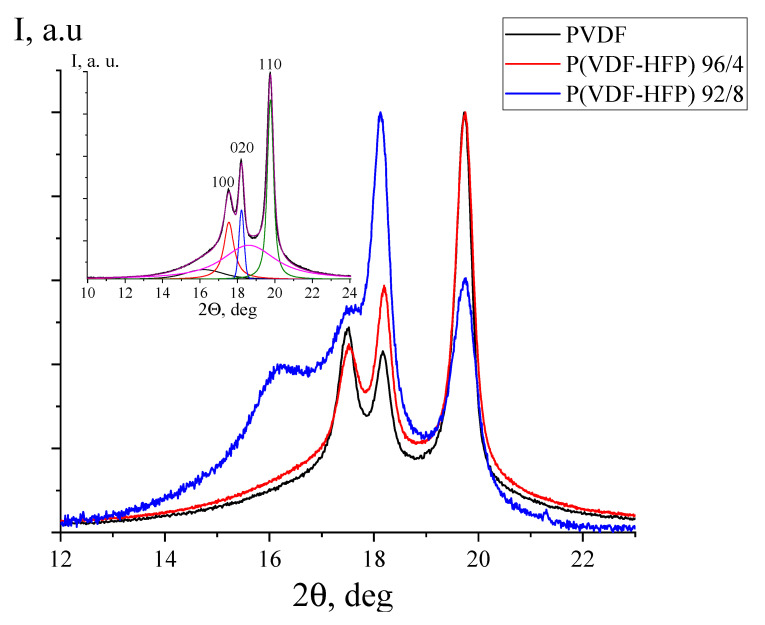
Comparison of X-ray diffraction curves for PVDF films, VDF/HFP copolymer of composition 96/4 and VDF/HFP copolymer of composition 92/8; the inset is an example of splitting the curve of the VDF/HFP copolymer of composition 96/4 into components for the calculation of the X-ray degree of crystallinity.

The inset to Figure 10 shows an example of the separation of overlapping reflections, from which the crystallinity *χ* of the films was calculated. Its variation with the fraction of HFP in the copolymer is shown in Table 4. It decreased naturally with the increasing content of the marked co-monomer. The crystal sizes along different directions were calculated from the half-width of the separated reflections according to Equation (1). It follows from Table 3 that these sizes also decrease with an increase in the fraction of HFP.

The structure of peak–halo could be inferred from the angular position of 2θ~16 degrees, which is often attributed to the amorphous phase. The noted peak–halo is observed on a number of other copolymers of HDF, for example with tetrafluoroethylene TFE or trifluoroethylene TrFE [24], which crystallize in the polar *β*-phase. Temperature X-ray diffraction images show that it should be attributed to the presence of residuals of a metastable (at room temperature, which is significantly below the Curie point) paraelectric phase. This can arise due to the manifestation of memory effects in the polymer melt or solution during the crystallization of the film under conditions of high supercooling [21,22]. For the considered copolymers, the noted peak-halo is observed at 2*θ*~18, i.e., the para-electric phase has less dense packing than the ferroelectric crystal [18,19,20].

Since the considered copolymers crystallize mainly in the nonpolar α-phase, the peak–halo with an angular position of 2*θ*~16 degrees (Figure 10) cannot be formally attributed to the paraelectric phase, since there is no “classical” ferroelectric *β*-phase. The specific structures of the polymers under consideration and the presence of kink-defect movements along the c-crystal axis in the α-phase create the possibility of forming domains in it as well, as recorded earlier by piezo-force microscopy [35]. The data of [36] also agree with this conclusion. Therein, the hysteresis curves on PVDF films crystallized in nonpolar α-phase were also observed using the classical method (Sawyer–Tower scheme). In this regard, the reflex halo with an angular position of 2*θ*~16 (Figure 10) can also be attributed to the manifestation of the paraelectric phase, since in it the packing density is lower than in the α-phase crystal. Separately, it should be emphasized that in VDF-HFP copolymers, the observed halo appears to be shifted by about two degrees towards small angles compared to VDF–TrFE copolymers [24]. This means that the packing density in the marked phase of the VDF–HFP copolymer appears to be lower than that of the latter copolymers. This could be explained by fact that the HFP group contains the -C-F_3_ bond, which for steric reasons prevents the dense packing of chains.

**Figure 11:**
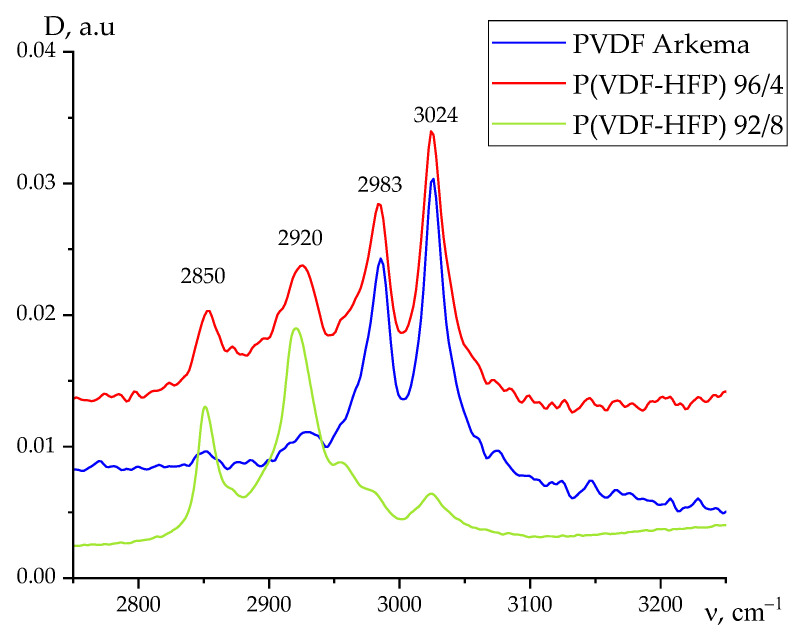
Comparative curves of ATR spectra in the valence vibration region of methylene groups for PVDF films, VDF/HFP copolymers of composition 96/4 and VDF/HFP copolymers of composition 92/8.

Figure 11 shows what changes in the structure of the considered phase as the HFP content in the considered copolymers increases. It can be seen that the increase in the proportion of HFP in the copolymer changes the ratio of intensities of characteristic bands 2920 and 3024 cm^−1^ This is quantitatively reflected in Table 4. As noted above, the ratio of intensities of the above bands can characterize the proportion of head-to-head defects in the film surface. It is evident from Table 4 that this should be the maximum for films of VDF/HFP copolymer of composition 92/8. This may be one of the reasons for the observed positive surface potential in such a film. Since in these films the share of regions with loose packing is the largest (Figure 10), it should be concluded that in less perfect crystals, favorable conditions are created for the escape of these defects into the surface. This chemical bonding defect contains two adjacent C–H bonds, wherein a positive charge is localized on the protons, since the electron density must shift to carbon. Perhaps this is one of the reasons for the observed positive surface potential in such a film [24]. At the same time, it can be noted that in such a copolymer, the proportions of areas with loose packaging are the largest (Figure 10). Therefore, it should be concluded that in this case, these defects should be displaced in the surface.

## 5. Conclusions

The influence of various factors on the formation of the surface potential of self-polarizing films is discussed via the example of a number of HDF copolymers. The problem of domain structure formation in the case of films crystallized in the nonpolar α-phase, which is poorly covered in the literature, is touched upon. In this connection, the question of the presence in the films under consideration at room temperature (much below the Curie point) of metastable paraelectric phase residues is raised. The physical prerequisites for this are memory effects in the melt or solution of the polymer, when the film is formed. It was found that the introduction into the PVDF chain of the HFP co-monomer with large steric hindrances during crystallization leads to the formation of a less densely packed paraelectric phase, and IR spectroscopy data show that this is accompanied by an increase in the probability of intrachain “head-to-head (tail-to-tail)” defects exiting to the surface.

## Figures and Tables

**Table 1:** The results of NMR for VDF/HFP 95.5/4.5.

Monomer Unit	Content, mol %
VDF normal	91.3
VDF reverse	4.2
HFP normal	4.2
HFP reverse	0.3

**Table 2:** Ratios of intensities of conformation-sensitive bands of ATR spectra and surface potential on the 1 and 2 sides of the VDP-tetrafluoroethylene copolymer film of composition 94/6 crystallized in β-phase.

Band, cm^−1^	442β430γ	470β490α	510β530α	1275β1235γ	Crystallinity	Surface Potential, V
					840cr905am	
Ratio side 12	5.44.8	0.870.56	6.36.1	0.90.7	10.612.9	0.40.7

**Table 3:** Ratios of intensity of conformation-sensitive bands of ATR spectra and surface potential on sides 1 and 2 of PVDF films and their copolymers, crystallized in the phase.

Bands, cm^−1^	410α430ggn	29203027	17201750	17201830	Crystallinity		Surface Potential, V
					765crα740am	975crα905am	
PVDF Ratio side 12	2.72.0	0.10.04	1.90.7	0.90.7	5.15.6	2.42.6	−2.1−0.2
P(VDF-HFP) side 12	2.72.3	0.60.5	1.30.9	0.31.2	4.85.6	1.92.1	−1.30.2

**Table 4:** Comparison of crystal sizes (*l_hkl_*), crystallinity *χ* and intensity ratio of *D*_2920_/*D*_3024_ absorption bands in PVDF copolymer P(VDF-HFP) films of different compositions.

Copolymer	l_c_ Crystal, nm			l_halo_, nm	χ	D2920D3024
	l_100_	l_020_	l_110_			
PVDF	17	26	22	3	0.49	0.1
P(VDF-HFP) 96-4	13	25	20	3	0.47	0.6
P(VDF-HFP) 92-8	7	23	16	4	0.40	4.2
